# Vocal biomarkers in heart failure—design, rationale and baseline characteristics of the AHF-Voice study

**DOI:** 10.3389/fdgth.2025.1548600

**Published:** 2025-05-02

**Authors:** Fabian Kerwagen, Maximilian Bauser, Magdalena Baur, Fabian Kraus, Caroline Morbach, Rüdiger Pryss, Kristen Rak, Stefan Frantz, Michael Weber, Julia Hoxha, Stefan Störk

**Affiliations:** ^1^ Department of Clinical Research and Epidemiology, Comprehensive Heart Failure Center, University Hospital Würzburg, Würzburg, Germany; ^2^ Department of Internal Medicine I, University Hospital Würzburg, Würzburg, Germany; ^3^ Department of Oto-Rhino-Laryngology, Head and Neck Surgery, University of Würzburg, Würzburg, Germany; ^4^ Institute of Clinical Epidemiology and Biometry, University of Würzburg, Würzburg, Germany; ^5^ Cosinuss GmBH, München, Germany; ^6^ ZANA Technologies GmbH, Karlsruhe, Germany

**Keywords:** heart failure, decompensation, digital health, telemonitoring, remote patient management, voice, vocal biomarker, study design

## Abstract

Acute heart failure (AHF) is a life-threatening condition and a common cause of hospitalization. The defining clinical feature of AHF is volume overload, leading to pulmonary and peripheral edema and consequently to weight gain. Vocal biomarkers have the potential to facilitate the early detection of worsening HF and the prevention of AHF episodes by offering a non-invasive, low-barrier monitoring tool. The AHF-Voice study is a prospective monocentric cohort study designed to investigate the trajectories of voice alterations during and after episodes of AHF, identify potential vocal biomarkers, and enhance the understanding of the pathophysiological mechanisms underlying these voice changes. It will examine the characteristics and determinants of vocal biomarkers, analyzing their correlations with patients' clinical status and comparing them to alternative clinical parameters in HF. Further, it aims to determine whether specific vocal biomarkers can accurately map different HF phenotypes and assess their association with patient trajectories**.** The study phenotypes patients hospitalized for AHF at admission and discharge, and follows them for a period of 6 months. During hospitalization, daily voice recordings are collected using a specially-designed smartphone app. Following discharge, patients are requested to continue daily voice recordings with their own smartphone for the subsequent six months the 6-month follow-up. Patient-reported outcome measures and body composition are assessed in the hospital and at follow-up visits. Sub-studies explore vocal fold oscillation through video-laryngostroboscopy and assess the feasibility of combining voice analysis with in-ear sensor technology for comprehensive digital phenotyping. A total of 131 patients were enrolled between April 2023 and November 2024: their mean age was 75 years (SD 10), 31% were women, 86% were in NYHA functional class III or IV, and 38% presented with *de novo* heart failure. Additionally, 59% of participants owned smartphones. The AHF-Voice study will provide insights into the potential of vocal biomarkers as reliable indicators of congestion, paving the way for innovative and accessible tools to support heart failure management.

## Introduction

### Rationale

Heart failure (HF) affects over 64 million individuals globally, with symptoms like breathlessness, fatigue, and edema (1). In Germany, acute decompensated HF is the most common reason for hospitalization (2). The in-hospital mortality of patients with acute HF (AHF) ranges between 4% and 10%, and mortality within the first year after discharge between 20% and 30% (3). Each hospitalization due to decompensated HF increases the risk of subsequent events (4). It is therefore imperative that incipient decompensation is promptly identified to prevent hospitalization (3, 5).

Patients with worsening HF usually experience a gradually increasing volume overload, accompanied by body weight gain and symptoms like dyspnea, peripheral edema and fatigue. Without adequate treatment, the patient will eventually require hospitalization due to further deterioration. The international HF guidelines recommend that patients assess their body weight daily as a primary non-invasive self-monitoring measure (3). In addition, non-invasive and invasive remote patient management (RPM) including hemodynamic monitoring of pulmonary artery pressure or self-monitoring weight measurement, has demonstrated beneficial effects (6–8). However, RPM require either invasive procedures, and/or the acquisition of additional hardware. There is a paucity of non-invasive alternatives for self-monitoring and reliable identification of incipient decompensation.

In recent years, advancements in digital technologies including artificial intelligence have facilitated the utilization of the human voice as a vocal biomarker for the diagnosis and management of diseases (9). Previous studies have reported the use of vocal biomarkers in neurodegenerative, cardiovascular and respiratory diseases (10–12). In the case of HF, Murton et al. showed that weight loss following diuretic therapy in patients hospitalized for AHF was associated with changes in specific voice features (13). Maor et al. demonstrated that vocal biomarkers can be generated from unstructured telephone-based recordings by crosslinking selected voice features with clinical outcomes. In their study, vocal biomarkers were associated with an increased risk of hospitalization and mortality in patients with congestive HF (14). The use of smartphone-based voice recordings and the automated generation of clinically meaningful vocal biomarkers reflective of changes in clinical status in patients with HF has been demonstrated by Amir et al. (15, 16). However, the evidence for vocal biomarkers in patients with HF is scarce and heterogeneous, with most studies encompassing small sample sizes and are limited to remote data collection after discharge.

Furthermore, the underlying pathophysiological mechanisms of voice alterations in patients with HF remain poorly understood. Several hypotheses are being discussed. (1) Murton et al. suggested that AHF-related volume overload leads to vocal fold edema that may cause changes in phonation, such as a hoarse voice with a limited vocal range, and leads to a changed fundamental and more irregular frequency (higher jitter) (13); (2) a general swelling of the vocal tract was proposed by Amir et al. (15, 16); that may influence formant frequencies (resonance characteristics) and contribute to reduced vocal clarity or shifts in frequency bands (17); (3) Reddy et al. showed that congestion alters sound pressure levels, which might be indicative for impaired lung function (18) relating to a reduced phonation duration and variability in intensity; (4) dysphonia has been observed in patients with left atrial enlargement, which may be caused by compression of the recurrent laryngeal nerve, known as cardiovocal or Ortner's syndrome, with the inability to control strengthening of the vocal folds and resulting in a hoarse and unstable voice (19, 20). Also, a combinations of these alterations are plausible mechanisms, as voice production involves an interplay between vocal fold oscillation and airflow (21).

The AHF-Voice study aims to improve the understanding of vocal biomarkers in HF by exploring the characteristics of voice alteration in patients with AHF via daily voice recordings throughout their in-hospital period of decompensation-recompensation and the post-discharge period thereafter. Additionally, it will assess the self-perceived measurements of voice alteration and anatomically visual differences in the state of decompensation and recompensation.

### Main research questions

The “Acute Heart Failure Voice Analysis Prospective Cohort Study” (AHF-Voice study) aims to explore the relationship between congestion and voice alterations in patients with AHF, with a view to reflecting their clinical condition. The study will address the following key questions: (1) What are the characteristics and determinants of vocal alterations in patients with AHF? (2) To what extent do these vocal alterations correlate with the patient's clinical status during an AHF episode? (3) To what extent are vocal biomarkers sensitive to changes over time, and how do they compare to established clinical parameters in HF such as quality of life or NT-proBNP levels? (4) Can specific vocal biomarkers or combinations thereof be mapped to different HF phenotypes? (5) Are vocal biomarkers associated with patient prognosis? (6) Are voice alterations in patients with AHF associated with pathophysiological changes, such as vocal fold edema, that affect vocal fold oscillation?

## Methods

### Study design

The AHF-Voice study is designed as a prospective monocentric observational cohort study. The study is being conducted at the University Hospital Würzburg as part of the UNISONO project, which is funded by the German Federal Ministry of Education and Research (Grant #16SV8877). The study was approved by the local Ethics Committee (245/22-me), complies with the Declaration of Helsinki and adheres to the STROBE reporting guidelines (22). The AHF-Voice study was registered at ISRCTN registry (ISRCTN13093083).

### Setting

**Figure 1:**
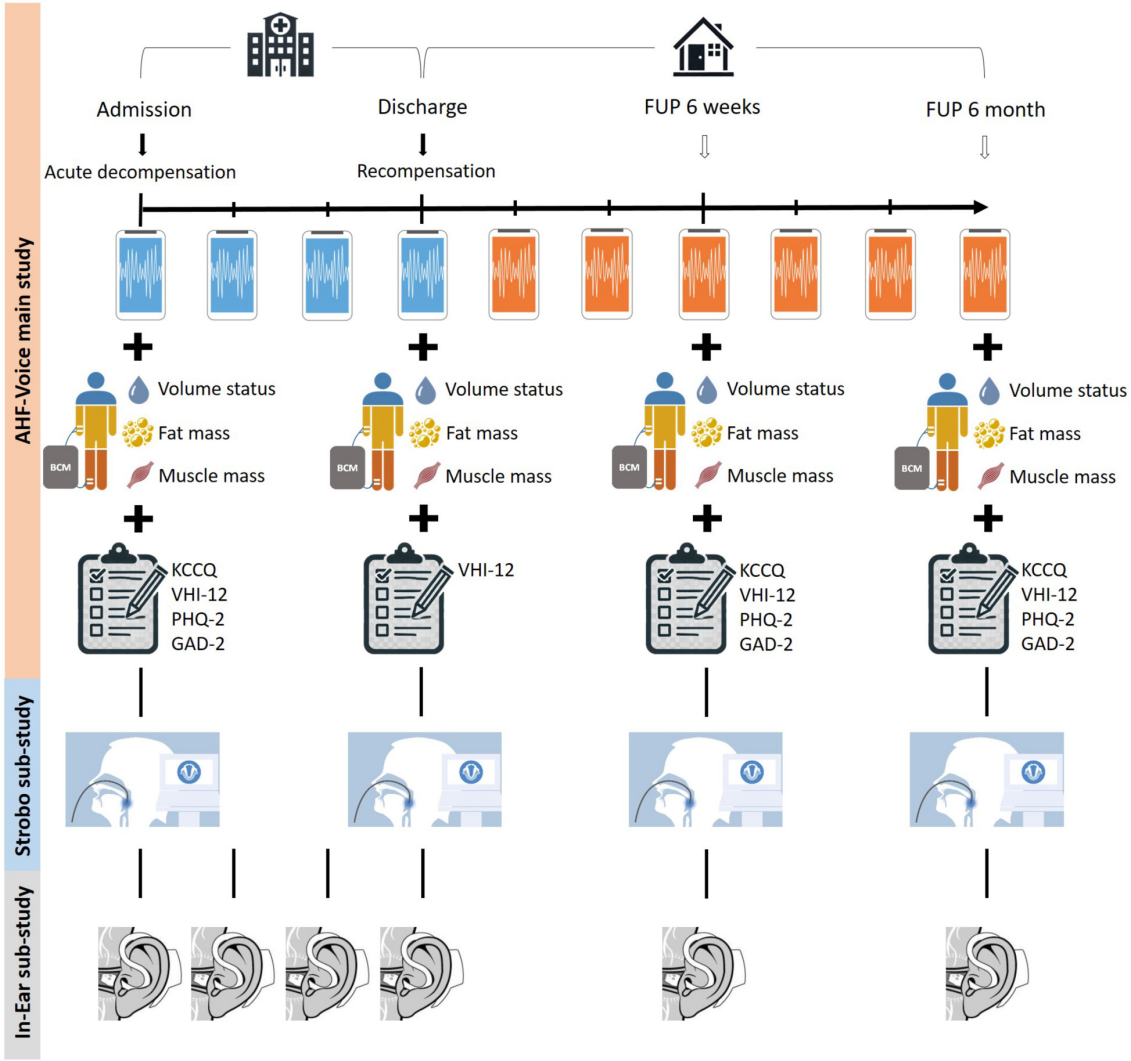
Design of the AHF-Voice main study and its two sub-studies. The figure shows the data collection over time. In the main study, daily voice recordings are performed during index hospitalization (blue smartphone), and voice recordings are continued at home on the patients’ own smartphones, if available (orange smartphone). In addition, body composition analyses and patient-related outcome measurements are carried out during admission, discharge, and follow-up visits. In the Strobo sub-study, a video-laryngostroboscopy and a voice field measurement will be performed on admission, discharge, and follow-up time points. As part of the In-ear sub-study, the in-ear sensor is used daily during index hospitalization, and at the 6-week and 6-month follow-up visits. BCM, body composition monitor; GAD-2, generalized anxiety disorder-2, KCCQ, Kansas City cardiomyopathy questionnaire; PHQ-2, patient health questionnaire-2.

All patients hospitalized at the Department Internal Medicine I of the University Hospital Würzburg are screened for study participation. The study period will be 6 months after index hospitalization, including structured follow-up visits after 6 weeks and 6 months. Figure 1 shows the study design. In addition to the primary study, two sub-studies will be conducted: the *Strobo* sub-study and the *In-Ear* sub-study.

### Participants

Inclusion criteria are: hospitalization with AHF (diagnosis compatible with international guidelines including edema, dyspnea, fatigue, and/or signs of congestion in chest x-ray and elevated NT-proBNP levels) (3), age ≥18 years, life-expectancy ≥6 months, willingness to attend planned follow-up visits at the outpatient clinic, and written informed consent. Exclusion criteria are: high output HF, cardiogenic shock, high-urgency listing for heart transplant, left ventricular assist device implanted/planned, and history of vocal fold disorder or vocal fold surgery.

### Index hospitalization

**Figure 2:**
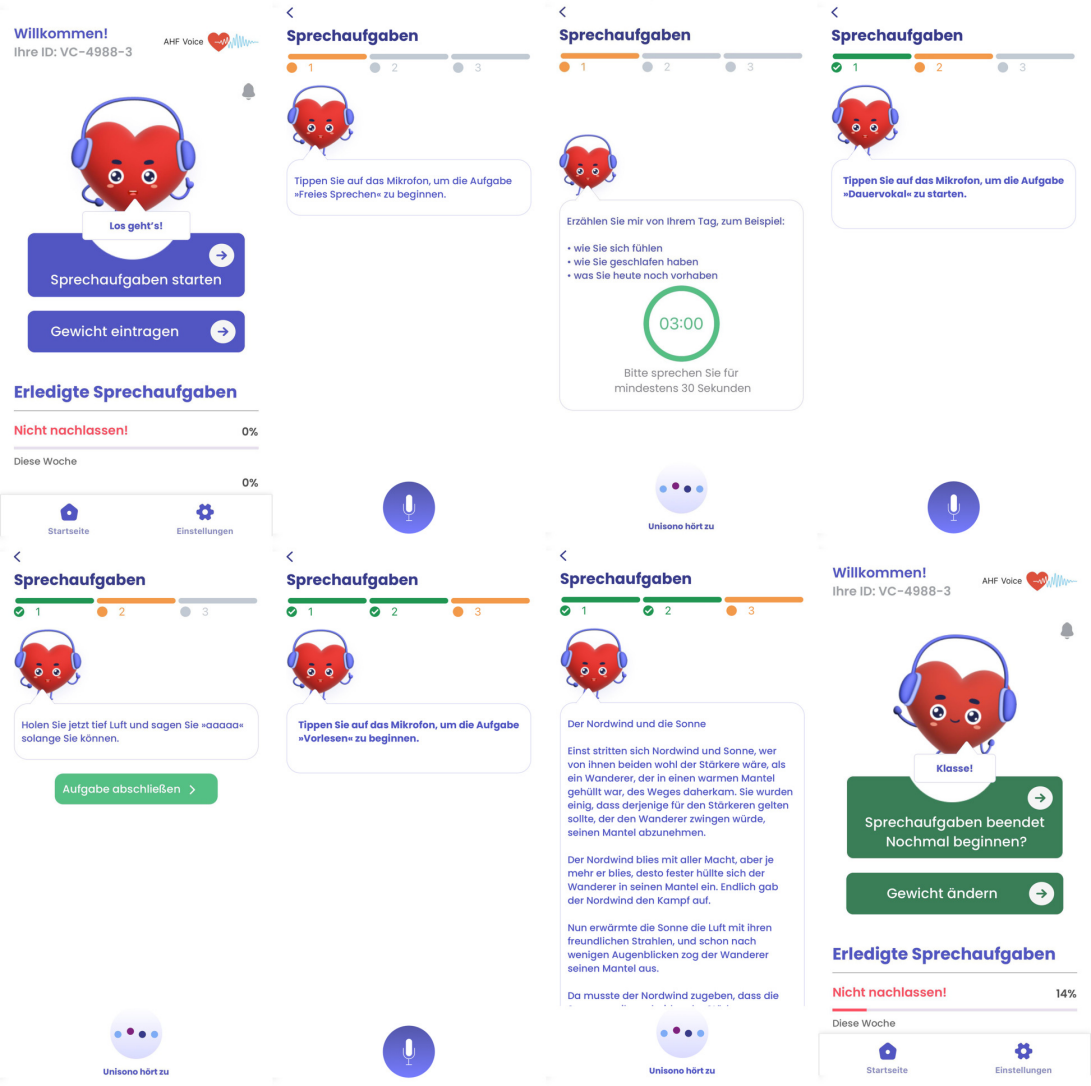
User interface of the specifically designed smartphone application in the AHF-Voice study. The application facilitates the recording of three distinct voice tasks: (1) spontaneous speech, (2) sustained vowel, and (3) reading of a text passage, one after the other. Additionally, the application allows the patient to maintain a weight diary. Screenshots from AHF Voice app, Zana Technologies GmbH.

During the index hospitalization, detailed phenotyping of each patient will be conducted, including medical history, clinical data (e.g., transthoracic echocardiogram, electrocardiogram), and routine blood tests. At index hospitalization, patients will complete quality of life self-report questionnaires regarding health-related quality of life (23-item Kansas City Cardiomyopathy Questionnaire) (23), depression (2-item Patient Health Questionnaire) and anxiety (2-item Generalized Anxiety Disorder) (24). Additionally, a voice impairment-related questionnaire (12-item Voice Handicap Index) (25) will be conducted at admission and discharge. Daily voice recordings will be collected using a specifically designed smartphone app (ZANA Technologies GmbH, Karlsruhe, Germany) and will be performed under supervision of study staff (see Figure 2). A comprehensive assessment of body composition (Body Composition Monitor, Fresenius Medical Care, Bad Homburg, Germany) is conducted at admission and discharge.

In addition to the daily voice recordings, patients contributing to the *In-Ear* sub-study, will explore the feasibility of using novel in-ear sensor technology for comprehensive digital phenotyping in patients with HF. Therefore, patients will have an in-ear sensor inserted and will wear it for at least 5 min daily, during which vital parameters will be recorded (*In-Ear* sub-study). The *Strobo* sub-study will focus on visualizing the pathophysiological aspects of vocal changes during the AHF period. For this purpose, patients will undergo video-laryngostroboscopy (video nasopharyngoscope XN HD, Xion Medical, Berlin, Germany) and voice field measurements (Digital Video Archive Software DiVAS; Xion Medical, Berlin, Germany) at admission and discharge as well. The obtained video and audio materials will be analyzed by a blinded phoniatrist.

### Follow-up period

All patients are asked to continue with the use of the mobile application for daily voice recordings after discharge on their own smartphone, if available. Six weeks and 6 months after the index hospitalization, patients are invited to the outpatient clinic of the Comprehensive Heart Failure Center at the University Hospital Würzburg (26). There, they will undergo standardized routine clinical re-evaluation including electrocardiogram, transthoracic echocardiogram, six-minute-walk test, pulmonary function tests, body composition analysis and video-laryngostroboscopy/voice field measurement (*Strobo* sub-study) and in-ear vital parameter measuring (*In-Ear* sub-study). Furthermore, participants will be asked to complete questionnaires regarding health-related quality of life (Kansas City Cardiomyopathy Questionnaire-23) (23), depression (Patient Health Questionnaire-2), anxiety (Generalized Anxiety Disorder-2) (24) and voice impairment (Voice-Handicap-Index-12) (25). Potential hospitalizations since the last study visit will be documented and tracked by requesting discharge letters. In case of death between follow-up visits, the cause will be clarified using death certificates, hospital letters, or reports from physicians and relatives. If patients do not attend follow-up visits, the study staff will conduct a standardized telephone-based interview to collect an abbreviated clinical data set.

### Surrogates of congestion

AHF-Voice utilizes a number of surrogate markers to assess the level of congestion. Daily weight recordings are taken during hospitalization and follow-up visits. Patients with their own smartphone can log their daily weight through app-based entries. Routine laboratory parameters, including NT-proBNP levels, are measured at admission and discharge. Additionally, body composition assessments will be performed at admission, discharge, and during follow-up visits, providing insights into fluid overload.

### Smartphone application

The smartphone application has been developed specifically for voice recording in the study. The app is available for iOS and Android systems and was developed through an iterative process that actively involved patients with HF in dedicated focus groups. Their feedback was incorporated into the design of the user interface to create a user-friendly and easy-to-use application (see Figure 2).

During index hospitalization, all patients are asked to perform daily voice recordings with a study smartphone (iPhone SE 2023, Apple Inc., Cupertino, USA) under the supervision of the study staff. Patients are then prompted through a voice dialogue interface on the smartphone app to perform the following set of speech tasks:

• Sustained vowel phonation (vowel/a:/)
• Spontaneous speech
• Reading standardized passage (“Northwind and Sun”)

During index hospitalization, study staff ensures that the same time of day is chosen for the daily voice recordings (e.g., 30 min–1 h after waking up). After discharge, patients will be asked to continue using the smartphone app on their own smartphone, if available. These patients will be instructed to install the smartphone app on their device prior to discharge, and will then be prompted to perform the speech tasks once a day. In addition, the daily weight measurements can be entered into the app. During the follow-up period, patients who do not own a smartphone will not collect voice recordings after discharge, but will contribute to the data set when attending the outpatient visits after 6 weeks and 6 months.

### In-ear sensor

**Figure 3:**
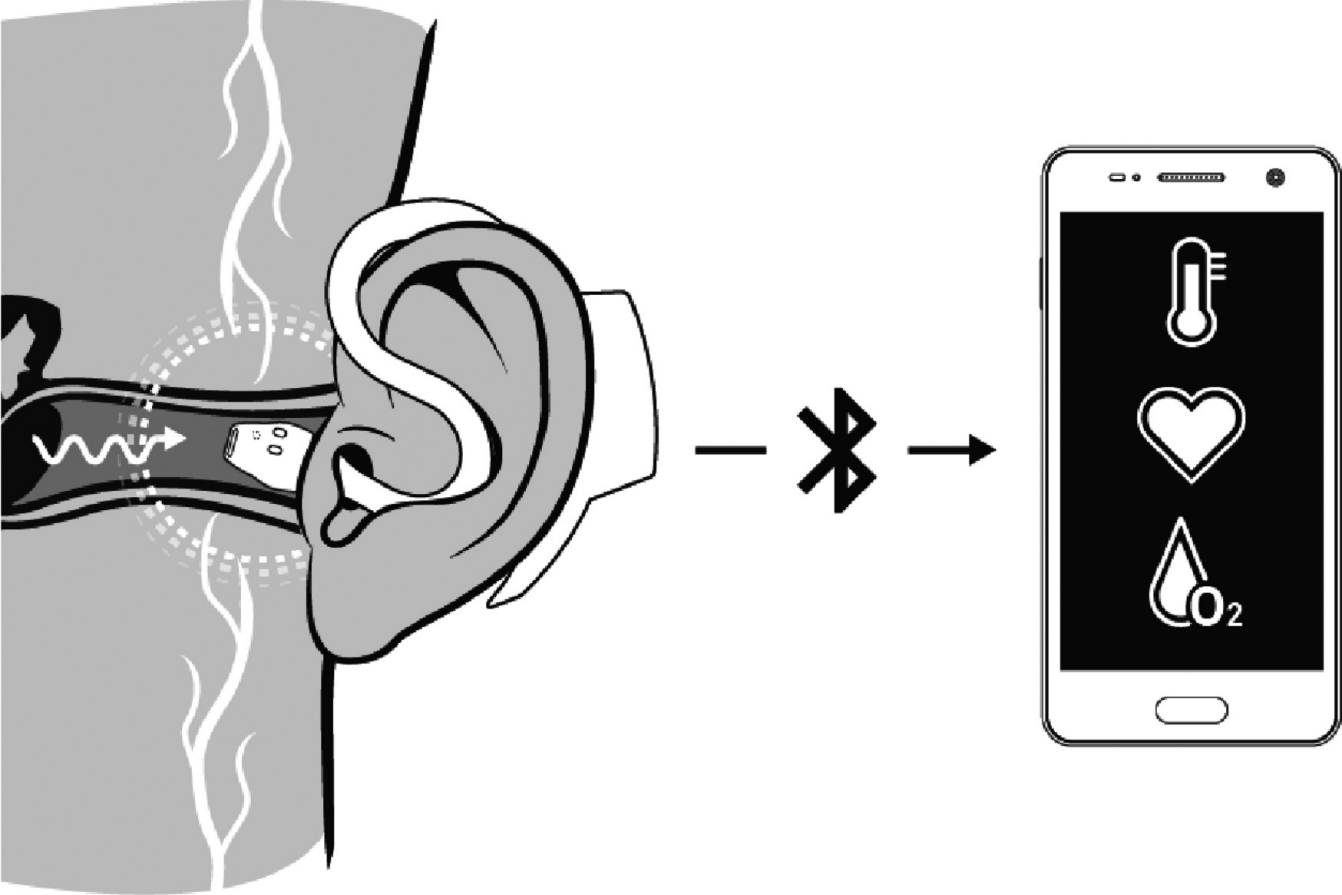
Application principle of the c-med° alpha in-ear sensor. The illustration shows the functionality of the in-ear sensor and illustrates its position within the ear canal. The data, which includes the pulse rate, oxygen saturation, and body temperature, is transmitted to a smartphone via bluetooth. Reproduced with permission from “Application principle of the c-med° alpha” by Cosinuss GmbH.

The in-ear sensor called *c-med° alpha* (Cosinuss GmbH, Munich, Germany) is comparable to a hearing aid and is inserted into the ear canal (Figure 3). It continuously and non-invasively measures body temperature, pulse rate, and blood oxygen levels in real time using optical sensors. It is approved as a Class IIa medical device (27, 28).

### Premature termination of follow-up

Withdrawal of consent will trigger premature termination of follow-up. Reason for withdrawal will be documented, and efforts will be made to complete the clinical information for this last patient contact. In case a patient misses a follow-up visit, study staff contact the patient directly in order to motivate him/her to attend the clinical follow-up visit at the Comprehensive Heart Failure Center (CHFC). If a visit at the CHFC isn't possible, telephone follow-up assessments and/or completion of background information via the general practitioner or other care providers will be attempted.

### Sample size calculation and power analysis

In general, there is no consensus about how to determine the sample size for clinical prediction models when applying AI methods (29). Also, the variability of sample size in the above-mentioned studies for voice analysis in patients with heart failure is high: whereas Amir et al. investigated voice alterations in 40 hospitalized patients for the association of vocal biomarkers with the binary status of congestion (“wet” vs. “dry”) (15), Maor et al. used the voice recordings of 10,583 patients (*n* = 8,316 for training cohort and *n* = 2,267 for test cohort) for the correlation of speech measures with hard clinical endpoints (i.e., hospitalization and death) (14). To conclude, the feasibility of vocal biomarker generation in patients with heart failure has been proven, but the optimal generation process is far from being well described.

The sample size calculation was performed using GPower software and based on the study of Amir et al. (16). In their study, the vocal biomarker (unitless value; mean value ± SD) of chronic heart failure patients undergoing hemodialysis treatment due to volume overload changed from 0.87 ± 0.17 before dialysis to 1.07 ± 0.15 after dialysis. The mean loss of body weight was 2 kg. From own results, we know that the volume reduction in hospitalized AHF patients is even greater (about 3 kg body weight) (30). Based on a repeated measures ANCOVA using the covariates left ventricular ejection fraction (LVEF ≥50% and <50%) and sex (♀, ♂) and assuming a conservative large variance (SD 0.30) and an effect size of *f* = 0.40, 111 patients are needed to achieve a power of 80% at alpha of 5%. Based on previous study experience, a drop-out rate of 10% was assumed. We therefore aim for a total sample size of 123 patients, which also allows for the analysis of changes in NT-proBNP using the methods described in Sahiti et al. (30). To this number were added those patients who withdrew from the study prematurely while still in hospital, resulting in a cohort of 131 patients.

### Data analysis of baseline characteristics

Data are summarized using descriptive statistics. For continuous variables, normally distributed data are presented as mean and standard deviation, while non-normally distributed data are expressed as median and quartiles. Categorical variables are reported as absolute numbers and percentages.

### Future statistical analyses

Data will be described using conventional methods according to the nature of the data. Repetitively sampled data will be investigated using generalized estimating equations (GEE) for repeated measures analysis (vocal feature) or repeated measures analysis of (co)variance (rANCOVA; e.g., for clinical data). To investigate the prognostic utility, Kaplan–Meier plots for graphical inspection and uni- and multivariable Cox proportional hazards regression analyses will be used. Subgroup analyses will include age, sex, *de novo* vs. decompensated chronic HF, heart failure with reduced ejection fraction (HFrEF) vs. heart failure with preserved ejection fraction (HFpEF). *post-hoc* comparisons of baseline findings and follow-up patient outcomes may be performed in the entire study and sub-populations of special interest. Appropriate adjustment for potential confounding and multiple testing will be considered for each research question separately.

AI-based methods, including machine learning and deep learning will be performed for the derivation of clinically meaningful vocal biomarker in patients with AHF applying primarily a binary approach (“decompensated” vs. “recompensated”).

### Missing data

All patients of the AHF-Voice study will be included, possibly with censored observation times. All efforts will be taken to prevent missing values in subjects who are alive. Whenever there is the possibility of significant bias due to missing data, additional analyses using imputation techniques will be employed and used for supportive sensitivity analysis.

## Results

### Recruitment and characteristics of the AHF-voice study sample

Between April 2023 and November 2024, 131 patients were recruited for the AHF-Voice Study. 50 (38%) of the patients participated in the *Strobo* sub-study, and 31 (24%) in the *In-Ear* sub-study. Of 131 patients, 9 (7%) of patients withdrew their consent during the follow-up observation time. Non-participation of patients was primarily attributable to physical or mental limitations.

Table 1 summarizes the baseline characteristics of the study population. The mean age of participants was 75 ± 10 years, 31% were women, and 38% were diagnosed with *de novo* HF. The majority of patients were in New York Heart Association (NYHA) functional class III (57%) or IV (29%) and the median NT-proBNP level at admission was 5,215 [2,650; 13,558] pg/ml. The most frequent underlying cause of HF was an ischemic etiology (42%), the mean LVEF was 47 ± 17, and 47% exhibited HF with a preserved ejection fraction (HFpEF). The most common comorbidities were diabetes (38%), peripheral artery disease (17%), chronic obstructive pulmonary disease (18%), and 10% current-smokers and 50% former smokers. The median length of hospital stay was 10 [7; 15] days. The majority of patients (59%) had their own smartphone, which enabled them to continue with daily voice recordings after discharge.

**Table 1:** Baseline characteristics of the AHF-Voice study cohort (*n* = 131).

Characteristic	Value
Sociodemographic	
Age (years)	75 ± 10
Women	40 (31)
Heart failure characteristics	
NYHA functional class (III/IV)	112 (86)
Ischemic heart failure etiology	55 (42)
*De novo* heart failure	50 (38)
Left ventricle ejection fraction (*N* = 127)	
≤40%	48 (37)
41–49%	17 (13)
≥50%	62 (47)
Comorbidities	
Atrial fibrillation	49 (37)
Peripheral artery disease	22 (17)
Diabetes mellitus	50 (38)
Arterial hypertension	117 (89)
Chronic obstructive pulmonary disease	23 (18)
Malignoma	14 (11)
Current smoker	13 (10)
Former smoker	65 (50)
Medical history	
Percutaneous coronary intervention/bypass	46 (35)
Valvular replacement (interventional/surgical)	19 (15)
Pacemaker or defibrillator	33 (25)
Measurements	
Body mass index (kg/m^2^)	30 ± 7
Systolic blood pressure (mmHg)	133 ± 26
NT-proBNP (pg/ml)	5,215 [2,650; 13,558]
eGFR—CKD-EPI (ml/min/1.73 m^2^)	44 [35; 70]
C-reactive protein (mg/dl)	1.0 [0.4; 2.5]
Patient-reported outcome	
Visual analog scale (*N* = 128)	40 [25; 50]
KCCQ-overall summary score (*N* = 124)	38 [27; 53]
PHQ-2 (score 0–6) (*N* = 119)	1 [0; 2]
GAD-2 (score 0–6) (*N* = 119)	0 [0; 1]
Patient characteristics	
Own smartphone	77 (59)
Length of hospital stay (days)	10 [7; 15]
Voice recordings obtained during hospitalization	21 [15; 30]

Data are *n* (%), mean (SD) or median [quartiles], as appropriate.

eGFR- CKD-EPI, estimated glomerular filtration rate—chronic kidney disease epidemiology collaboration equation, GAD-2, generalized anxiety disorder—2; KCCQ, Kansas City cardiomyopathy questionnaire; NT-proBNP, N-terminal pro B-natriuretic peptide; NYHA, New York Heart Association Functional Class, PHQ-2, patient health questionnaire-2.

Overall, 3,072 voice recordings (1,024 per voice task) were collected during the hospitalization period of all 131 patients, corresponding to a median of 21 [15; 30] voice recordings (7 [5; 10] per voice tasks) per patient.

## Discussion

The AHF-Voice study has been designed to provide new insights into the etiology, characteristics, determinants, progression, and prognostic utility of vocal biomarkers in patients experiencing an episode of AHF. Smartphone-based daily voice recordings are used as a substrate to detect subtle vocal changes, which are then associated with conventional markers of congestion.

The AHF-Voice study cohort represents a well-phenotyped population of patients with AHF. It comprises a high proportion of patients with NYHA functional class III and IV at admission, indicating a substantial number of patients with significant congestion. The cohort includes a significant proportion of patients with *de novo* HF and an even distribution between heart failure with a reduced or mildly reduced ejection fraction and HFpEF. The profound differences between LVEF-based HF phenotypes (31) will allow for stratified analyses, thereby exploring potential voice-based differences between these HF subtypes. Furthermore, a substantial number of voice recordings were collected during the index hospitalization and will be collected during follow-up period, enabling the application of advanced artificial intelligence methodologies for predictive modeling.

Changes of vocal biomarkers over time and their determinants remain under-researched. Previous studies only suggested the existence of distinct vocal biomarker patterns in individuals exhibiting either congested or decongested states (13, 15). To address this gap, the AHF-Voice study will investigate the longitudinal trajectories of vocal biomarkers in patients with AHF through daily voice recordings, both during the course of their hospitalization and over a 6-month observation period. This approach will facilitate a comprehensive assessment of vocal changes in relation to disease progression and patient outcomes, thereby contributing to the development of more personalized and predictive care strategies for patient with AHF.

According to the European Laryngological Society (ELS) and the American Speech-Language-Hearing Association, a comprehensive voice assessment for general voice impairments in the field of phoniatrics is suggested (32, 33). The AHF-Voice study will be the first to comprehensively examine all four (auditory, visual, physician-reported, and patient-reported) dimensions of the human voice: (i) vocal characteristics are captured through the recording of various voice tasks; (ii) visual assessment of the vocal cords is conducted using video-laryngostroboscopy, with vocal fold description following the guidelines of the ELS (32); (iii) an independent phoniatrist evaluates the voice using the RBH (roughness, breathiness, hoarseness) scale; (iv) all patients provide self-reported outcomes via the Voice Handicap Index-12.

As previously stated, the pathophysiological reasons for voice alterations in patients with HF remain unclear. The considerable number of patients participating in the *Strobo* sub-study will enable the provision of innovative insights into the pathophysiological aspects of disturbed vocal cord function and its potential association with prognosis, including rehospitalization and mortality within six months. Furthermore, the quality of voice recordings can vary due to factors such as ambient noise, the distance between the mouth and microphone, and the type of smartphone used by the patient after discharge (34). To assess and compare these impacts, simultaneous voice recordings were collected using both a smartphone and the reference standard of voice field measurement.

The importance of digital health technology in clinical trials is increasing (35, 36). Our study encompasses the development of a dedicated mobile solution and deploys smartphone-based remote data collection, allowing for asynchronous and continuous data collection in both hospital and home settings. The results from the AHF-Voice study will inform future decentralized and virtual clinical trials. Furthermore, vocal biomarkers may serve as a novel digital endpoint in future HF trials.

The study has several limitations. First, selection bias may affect the generalizability of the findings, as only German-speaking participants were included, which limits the applicability of the results to non-German speakers. Further studies should be conducted in additional languages in order to gain a more comprehensive understanding of the impact of language. Alterations in voice quality resulting from upper respiratory infections during the study period could confound the analysis of vocal biomarkers. For this purpose, a longitudinal data collection approach was chosen, and clinical (e.g., fever) and laboratory parameters (e.g., C-reactive protein) of inflammation assessed at each study visit. Finally, it is possible that participants' increased awareness of potential voice disorders, due to their involvement in the study, may influence their self-assessments of their voice.

## Conclusion

The AHF-Voice study provides a comprehensive framework deriving vocal biomarkers from voice recordings, covering all four dimensions of voice diagnostics: self-reported outcomes, physician-reported assessments, visual evaluation of the vocal cords, and acoustic voice analyses. The longitudinal design of the study permits the continuous monitoring of the voice over time, thereby offering valuable insights into the trajectories of voice alterations and their association with disease progression and prognosis in patients with HF experiencing an AHF episode. Moreover, the investigation aims to explore the potential of vocal biomarkers as a future tool for telemonitoring, with the objective to enable the early detection of decompensation in HF patients. In addition, the AHF-Voice study will inform on the utility of vocal biomarkers as digital endpoints for future HF trials.

## Data Availability

The original contributions presented in the study are included in the article/Supplementary Material, further inquiries can be directed to the corresponding author.
